# OLDER PEOPLE AND TECHNOLOGY: THE GOOD, THE BAD, AND THE UGLY

**DOI:** 10.1093/geroni/igz038.768

**Published:** 2019-11-08

**Authors:** Toni C Antonucci

**Affiliations:** University of Michigan, Ann Arbor, Michigan, United States

## Abstract

The use of multiple technologies in the service of social relations in widely evident. It is not at all clear, however, that we recognize the fundamental changes in social relations that are occurring as a result. Some changes are quite positive, e.g., low cost maintenance of geographically distant but emotionally close relationships. Others can be quite negative, e.g., the lost ability to gauge emotional reactions through face-to-face contact, often resulting in unnecessarily hurtful behaviors. Preliminary data indicate that people selectively use different forms of communication under positive circumstances, e.g. to transmit good news; or negative circumstances, e.g. resolve a dispute/express anger and dependent on the nature or closeness of the relationship e.g., parent, spouse, child. We need to be mindful of the good, the bad, and the ugly of technology; and, its specific effect on the relationships of and with older people.

